# Transglutaminase in neurological disease

**DOI:** 10.18632/oncotarget.19694

**Published:** 2017-07-31

**Authors:** Guylaine Hoffner, Philippe Djian

**Affiliations:** Laboratoire de Physiologie Cérébrale, UMR 8118, Centre National de la Recherche Scientifique, Université Paris Descartes, Paris, France

**Keywords:** protein aggregation, polyglutamine, Alzheimer, Parkinson, prion

Transglutaminase catalyzes the formation of intermolecular isopeptide crosslinks in polypeptides [7]. Because the crosslink is insensitive to proteolysis, polymers extensively crosslinked by transglutaminase are extremely resistant. Thus keratinocyte transglutaminase is responsible for the formation of the insoluble envelope of the corneocyte, which protects the epidermis against chemical and physical injuries [8]. The stabilization of the blood clot, at the late stage of coagulation, is caused by the crosslinking of fibrin by factor XIII, a circulating transglutaminase [6]. Transglutaminase is abundant in the brain and particularly in neurons. The function of neuronal transglutaminase remains unknown, but a connection between the catalytic activity of transglutaminase and neurological diseases was proposed a while ago [2]. This connection rests in the glutamine residue, which is both a substrate for transglutaminase when in a protein and the cause of neurological disease when mutationally expanded.

Glutamine residues (usually encoded by CAG) are necessary for the formation of the crosslinks and most transglutaminase substrates contain a segment of short tandem sequence repeats [3]. A number of human diseases, such as Huntington disease, are associated with genes containing an excessive number of CAG reiterations and the associated diseases are primarily in the central nervous system. All the diseases of polyglutamine (polyQ) expansion are associated with insoluble protein aggregates in neurons [4]. It has been shown that as a result of excessive glutamine reiteration, the mutant proteins became substrates of transglutaminase and it has been postulated that the aggregates resulted from the crosslinking of the mutant proteins to other proteins that could act as lysyl donors [5]. Although the mutant proteins are widely expressed, there are several possible explanations for the neuronal specificity of their mutations: transglutaminases and their substrates, such as synapsin I, have a role in synaptic transmission; neurons are less able to degrade transglutaminase produced aggregates than other cell types; there is no renewal of neurons to compensate for cell lethality; or the transglutaminase becomes activated by the rise in Ca^2+^ that occurs during synaptic transmission [2].

In addition to diseases of polyQ expansion, neurological diseases associated with the formation of protein aggregates include Alzheimer’s disease, Parkinson’s disease, prion diseases and some forms of amyotrophic lateral sclerosis. All of these diseases are characterized by the deposition of specific proteins: the amyloid β-peptide and hyperphosphorylated tau in Alzheimer’s disease, ɑ-synuclein in Parkinson’s disease and the conformationally altered Prion protein in Prion disease. Numerous reports have provided supporting evidence linking neurological diseases and the formation of aggregates to the action of transglutaminase [3]. The role of transglutaminase has also been challenged. Recent data have suggested that protein aggregates are structurally diverse and that differences in structure might explain why some aggregates are toxic, neutral or even beneficial [4]. In addition to its stabilizing effect, transglutaminase-catalyzed crosslinking might induce structural changes in early aggregates thus altering their conformation and by way of consequence their toxicity [1].

In view of the likely participation of transglutaminase in neurodegenerative diseases and of the elusive function of transglutaminase in brain, we endeavored to identify the transglutaminase substrates present in brain thus hoping to clarify the function of the enzyme in brain. For this we developed a functional proteomics strategy in which incorporation of biotinylated amine-donor and amine-acceptor probes is used to affinity-purify the transglutaminase substrates, which are then identified by mass spectrometry. The biological significance of the 166 substrates that we found was determined using the Ingenuity Pathway Analysis. We were surprised to find that most of the brain substrates identified were known to interact with huntingtin, the amyloid precursor protein or α-synuclein and that neurological disease was the most significant canonical pathway associated with the substrates. In view of the likely association of transglutaminase substrates with neurological disease, we wondered whether the aggregates associated with the diseases contained such substrates. This was demonstrated in two ways. First transglutaminase promoted very efficiently the incorporation of the amine-acceptor probe into both the inclusions of Huntington disease brain, and huntingtin-containing polymers purified from a patient’s cerebral cortex. Second, we randomly selected some substrates and tested their presence within the inclusions by in-situ immunolabeling. All the substrates that we examined could be detected in the inclusions (Figure 1).

**Figure 1:**
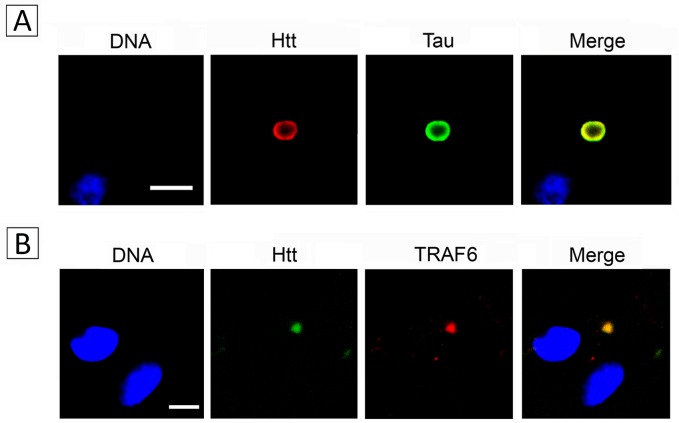
Tau and TRAF6, two substrates identified by functional proteomics, are found in the inclusions present in the brain of patients affected by Huntington disease Inclusions were double-stained with anti-N-terminal huntingtin and either anti-Tau (A) or anti-TRAF6 (B) antibodies. Sections were counterstained with TO-PRO-3 for DNA and examined by confocal microscopy. Scale bars: 5 μm.

Since the substrates that we had found had been identified in an *in-vitro* assay, it was necessary to demonstrate that they could also act as substrates in intact neuronal cells. For this we treated neuronal cells with molecules that increase cytosolic Ca^2+^ concentration and thus activate the latent transglutaminase activity found in these cells. Three substrates (actin, β-tubulin and a neurofilament subunit) were randomly selected for the experiment and all three were found to be selectively polymerized in neuronal cells when cytosolic calcium concentration was raised. These results strongly support the idea that the crosslinking activity of brain transglutaminase participates in the formation of the protein aggregates found in diseases of the central nervous system and reinforce the notion that transglutaminase might constitute a useful target in the search for prophylactic or therapeutic molecules inhibiting the aggregation process [1].

## References

[1] André W (2017). Neurobiol Dis.

[2] Green H (1993). Cell.

[3] Hoffner G (2010.). CNS Neurol Disord Drug Targets.

[4] Hoffner G (2015). Mol Neurobiol.

[5] Kahlem P (1998). Mol Cell.

[6] Laki K (1948). Science.

[7] Mycek M (1959).

[8] Rice R (1978). J Cell Biol.

